# Supplementary material for: Assigning Co-Regulated Human Genes and Regulatory Gene Clusters
Source: Cells. 2021 Sep 12;10(9):2395. doi: 10.3390/cells10092395 (PMC8470523; doi:10.3390/cells10092395)
Supplement: Supplementary file 1 [file cells-10-02395-s001.zip › supplementary.pdf]

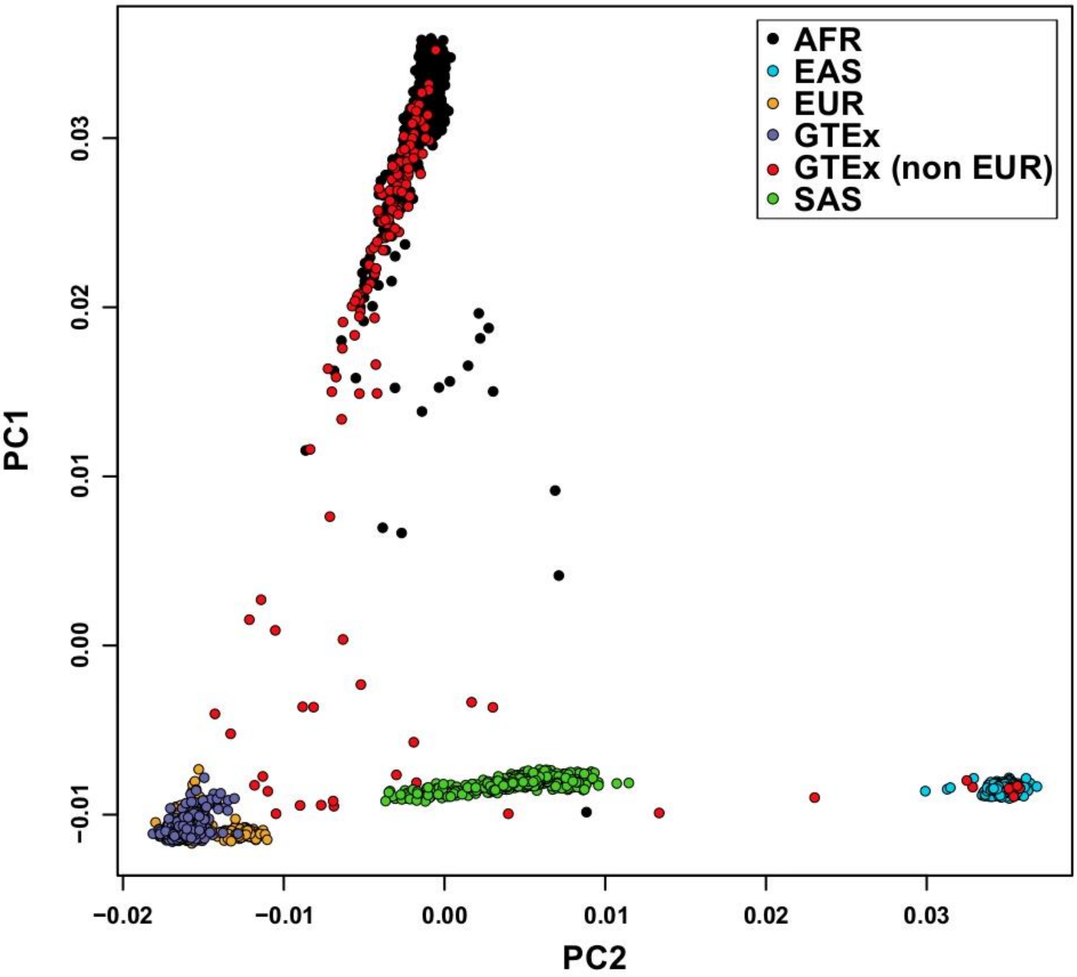

Figure S1. Genotype principal component analysis (PCA). 100,000 autosomal variants were randomly extracted from the GTEx dataset. In addition, the genotypes from samples of European (EUR, yellow), African (AFR, black), South Asian (SAS, green), and East Asian (EAS, light blue) ancestry were selected from the 1000 Genomes Project [27]. PCA was performed and the first two principal components (PC1 and PC2) of the 838 samples from the GTEx project (dark blue) were plotted. One hundred and forty-four of these samples (red) did not cluster next to the European reference samples and thus were excluded from further eQTL calculations.

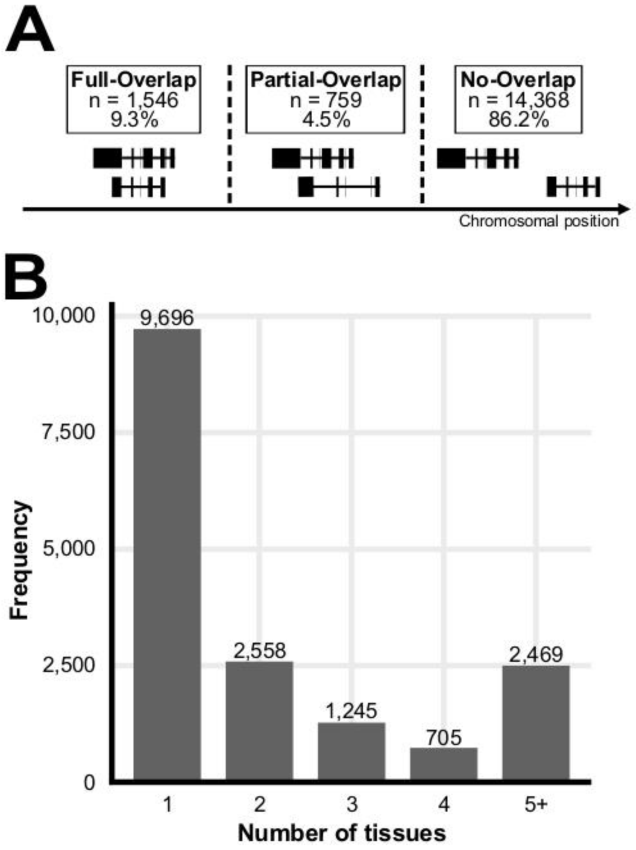

Figure S2. Characterization of colocalizing gene pairs. (**A**) Relative position of colocalizing genes on the chromosome. Altogether 16,673 unique colocalizing gene pairs were identified in the 24 tissues with a sample size above 200. In total, 1,546 of these gene pairs revealed full-overlap, 759 partial-overlap, and 14,368 no overlap. The mean distance of the genes in the last category was 122,265 bp (SD: 267,498 bp). (**B**) Numbers of shared colocalizing gene pairs across tissues. Of the 16,673 unique colocalizing gene pairs, 9,696 were exclusively present in a single tissue, whereas 2,469 gene pairs showed a colocalization in more than five (5+) tissues.
